# Differential proviral latency of the HIV-1 subtypes B and AE

**DOI:** 10.1186/1742-4690-8-S2-P72

**Published:** 2011-10-03

**Authors:** Renée M van der Sluis, Ben Berkhout, Rienk E Jeeninga

**Affiliations:** 1 Laboratory of Experimental Virology, Dept Medical Microbiology, Center for Infection and Immunity Amsterdam (CINIMA), Academic Medical Center, University of Amsterdam, The Netherlands

## 

The current HIV-1 pandemic is caused by at least nine subtypes (termed A through K) and an increasing number of recombinant forms. There has been a large skew in research focus on subtype B, which is the most prevalent subtype in the Western world. We study the transcriptional promoter in the long terminal repeat (LTR). Each subtype has a specific LTR promoter configuration and even minor changes in the transcription factor-binding sites (TFBS) or their rearrangement can have a significant impact on cell tropism and pathogenicity. For the HIV-1 subtypes, the LTR promoter variation could contribute to differences in proviral latency. Such differences in latency properties may have an impact on the establishment of viral reservoirs and the inability to clear the virus by therapeutic intervention.

Latency remains a formidable barrier towards virus eradication as therapeutic attempts to purge these reservoirs have been unsuccessful. The pool of latent proviruses is established early during infection and forms a steady source of proviral DNA that can last a lifetime in infected individuals.

We have demonstrated previously that there are differences in proviral latency properties between the subtypes, which are determined in part by the LTR promoter. Most interesting was the observation that subtype AE combined higher basal transcription (-Tat) with reduced proviral latency. The naturally occurring replacement of an NFkB for GABP site was partially responsible for this AE-specific latency phenotype. Another transcription factor that may contribute to the increased basal transcription of subtype AE is AP-1, which is present in subtype AE but absent in subtype B. We generated luciferase constructs with and without this AP-1 site in both subtype LTR backbones to determine the influence of AP-1. The luciferase constructs contain the core promoter region with all major TFBS (e.g. NFkB and Sp1 sites). Results obtained with these reporter constructs will be presented.

